# Modulation of liver glucose output by free or restricted feeding in the adult rat is independent of litter size

**DOI:** 10.1186/s12986-019-0413-0

**Published:** 2019-12-12

**Authors:** Laís Akemi Yamada, Isabela Ramos Mariano, Vanessa Lara Rissi Sabino, Renan Soares Rabassi, Camila Bataglini, Silvia Carla Santana Ferreira Azevedo, Nayra Thais Delatorre Branquinho, Mirian Ayumi Kurauti, Rosângela Fernandes Garcia, Maria Montserrat Diaz Pedrosa

**Affiliations:** State University of Maringá, Maringá, 87020-900 Brazil

**Keywords:** Caloric restriction, Litter size, Liver metabolism, Glycogenolysis, Gluconeogenesis, Metabolic programming

## Abstract

**Background:**

Caloric restriction since birth changes glucose metabolism by the liver in overnight-fasted rats to a fed-like pattern, in which glucose output is large but gluconeogenesis is negligible. It was investigated whether these changes could be a residual effect of the nutritional condition during lactation and what could be the mechanism of such change.

**Methods:**

Newborn *Wistar* rat pups were arranged in litters of 6 or 12 (G6 and G12). After weaning, the male pups were divided in: G6L and G12 L, fed freely until the age of 90 days (freely-fed groups); G6R and G12R, given 50% of the GL ingestion (food-restricted groups) until 90 days of age; G6RL and G12RL, given 50% of the GL ingestion until 60 days of age and fed freely until 90 days of age (refed groups). The experimental protocols were carried out at the age of 90 days after overnight fasting. Pairs of groups were compared through t test; other statistical comparisons were made with one-way ANOVA with Tukey post hoc text.

**Results:**

Caloric restriction was effective in decreasing body and fat weights, total cholesterol and LDL. These effects were totally or partially reversed after 30 days of refeeding (groups GRL). During liver perfusion, the high glucose output of the GRs was further enhanced by adrenaline (1 μM), but not by lactate infusion. In contrast, in groups G6L, G12 L, G6RL and G12RL glycogenolysis (basal and adrenaline-stimulated glucose output) was low and gluconeogenesis from lactate was significant. A twofold increase in liver content of PKA in group G6R suggests that liver sensitivity to glucagon and adrenaline was higher because of caloric restriction, resulting in enhanced glucose output.

**Conclusions:**

As glucose output was not affected by litter size, liver glucose metabolism in the adult rat, in contrast to other metabolic processes, is not a programmed effect of the nutritional condition during lactation. In addition, the increased expression of PKA points to a higher sensitivity of the animals under caloric restriction to glycogenolytic hormones, a relevant condition for glucose homeostasis during fasting.

## Background

Caloric restriction represents a challenge to an organism by limiting the supply of energy and nutrients. However, currently it has been one of the interventions aimed at delaying aging and its related illnesses, increasing the expectancy of a healthy life and most of all decreasing obesity and its comorbidities [1, 2]. Therefore, in experimental models, caloric restriction imposed to adult animals is often preceded by obesogenic diets or interventions [3, 4].

Whatever the purpose of caloric restriction, glucose homeostasis is a priority within the scope of energy metabolism, because it assures to the glucose-dependent tissues, such as the brain, a continuous and appropriate supply of this nutrient. Our research group has been investigating changes of systemic and liver glucose metabolism caused by caloric restriction in rats [5–8]. Caloric restriction since birth was chosen because it is a permanent limited supply of calories and nutrients capable of impairing not only growth, but also other morphological and physiological aspects, such as fat deposition, insulin sensitivity, glucose tolerance and energy metabolism.

A special attention has been given to glucose metabolism by the liver, the central organ of glucose homeostasis. So far, the results demonstrate that rats raised in litters with more pups and kept under 50% caloric restriction after weaning exhibit high liver glucose release and little or no additional glucose release in the presence of a gluconeogenic substrate, the opposite of what is expected after overnight fasting [5, 6, 8]. Later, experiments in which these rats were freely fed after caloric restriction [9] showed a partial reversal of the intense glucose release, suggesting that the liver response was linked to the present feeding regimen.

Several experimental studies and epidemiological surveys correlate the nutritional condition during lactation, both in rodents and humans, with metabolic derangements of later appearance (that is, in adult life), especially obesity, glucose intolerance, insulin resistance and other related impairments [10–12]; this is called metabolic programming. As the nutritional manipulations after weaning carried out by our research group were not investigated in rats from litters of fewer pups, we hypothesized that the alterations observed in liver glucose metabolism could be a residual effect of the nutritional restriction during lactation. Therefore, in this study it was investigated the liver glucose metabolism of male *Wistar* rats from two litter sizes subjected to different combinations of free feeding and 50% caloric restriction after weaning. Additionally, a possible mechanism could be proposed for the altered liver glucose metabolism caused by caloric restriction.

## Methods

The experimental procedures were approved by the Ethics Commission on Animal Use (CEUA certificate 8401200317 of May 9, 2017) of the State University of Maringá (UEM) and followed the principles on animal experimentation of the National Council of Control on Animal Experimentation (CONCEA, Brazil).

### Experimental groups

Pregnant *Wistar* female rats were obtained from the Central Animal House of UEM. The dams and their litters, as well as the experimental groups, were kept at the animal house of the Department of Physiological Sciences under controlled illumination (12 h light/12 h dark), temperature (22 ± 2 °C) and air exhaustion. The dams were given free access to rodent chow and water during gestation and lactation. One day after delivery, the litters were arranged to either 6 or 12 pups (G6 and G12 litters, respectively), preferably males. Female pups were kept only when necessary to complete litter size.

**Fig. 1:**
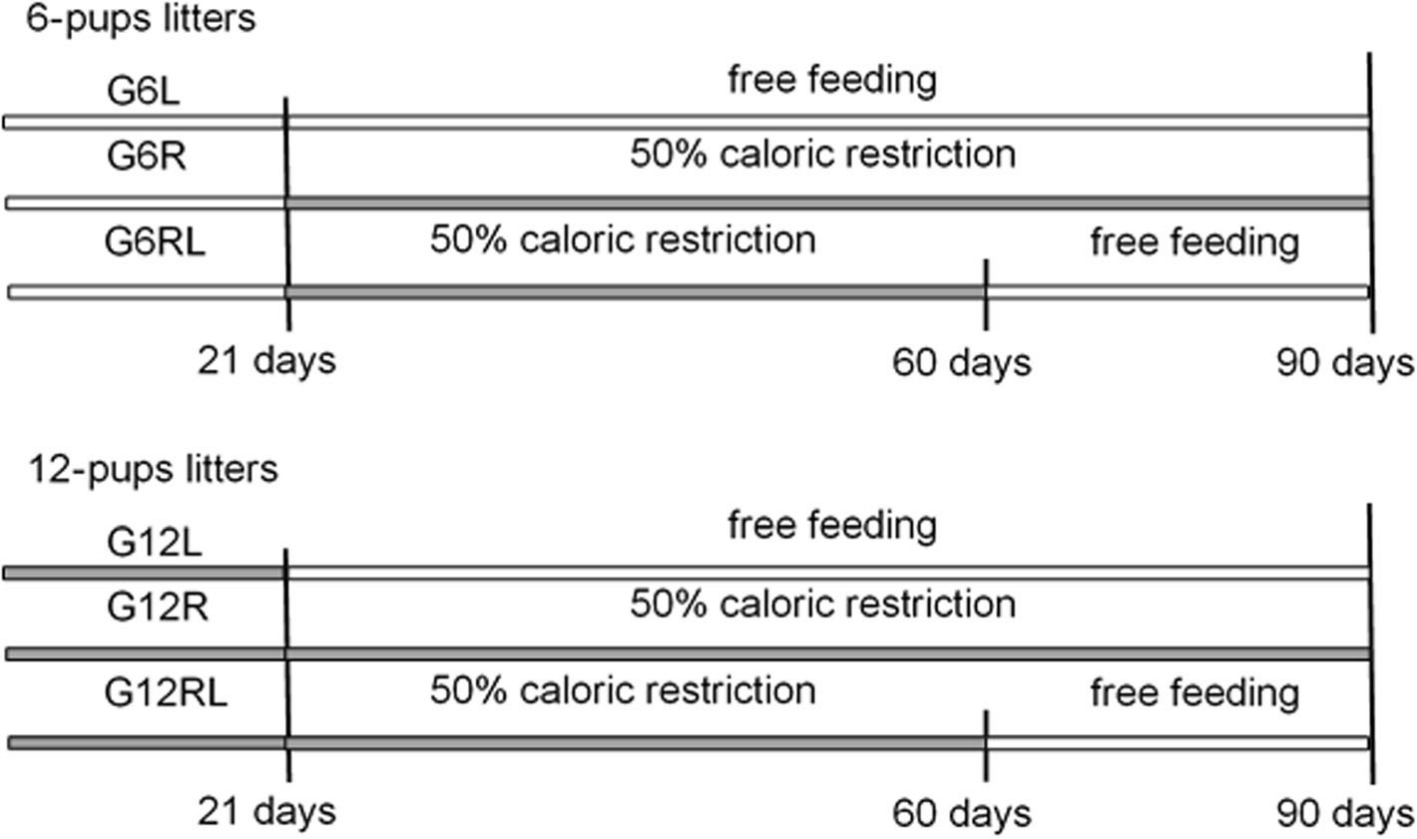
Representation of the experimental design of the groups

At weaning (21 days after birth, 21 d), the dams and female pups were given an i.p. anesthetic overload of thionembutal 120 mg/kg after lidocaine 5 mg/kg for euthanasia. The male pups were put in plastic boxes in groups of 3 according to original litter size and post-weaning feeding regimen. The rats from the 6-pups litters composed the groups G6L (fed freely from weaning until 90 d), G6R (subjected to 50% caloric restriction relative to the amount eaten by the G6L from weaning to 90 d) and G6RL (subjected to 50% caloric restriction relative to the amount eaten by the G6L from weaning to 60 d, then fed freely until 90 d). The rats from the 12-pups litters composed the groups G12 L, G12R and G12RL, subjected to the same feeding regimens described for G6L, G6R and G6RL, respectively. The experimental groups are illustrated in Fig. 1.

The standard rodent chow (Nuvilab, Curitiba-PR, Brazil) consisted of calcium carbonate; corn, soybean, and wheat bran; bicalcium phosphate; and premix of vitamins, minerals, and amino acids; and levels were 12.5% humidity, 22% raw protein, 4% ethereal extract, 10% mineral matter, and 8% fibrous matter.

The experimental procedures were carried out at the age of 90 d after overnight fasting (approx. 14 h).

### Removal of biological material

Six animals of each group were given an i.p. injection of thionembutal 120 mg/kg after lidocaine 5 mg/kg. Blood and liver were rapidly removed. A blood sample was immediately used to determine fasting blood glucose with test-strips and glucometer (Optium Exceed®; Abbott, São Paulo-SP, Brazil); another blood sample was centrifuged at 4000 rpm for 5 min. Total and HDL cholesterol and triglycerides were determined on the serum (commercial kits GoldAnalisa, Belo Horizonte-MG, Brazil). Values of LDL and VLDL were estimated with the equations: VLDL = triglycerides/5; LDL = total cholesterol – (HDL + VLDL) [13]. All data were expressed as mg/dL.

The liver was weighed, immediately immersed in liquid nitrogen and stored at − 80 °C for further analyses.

### Determination of liver content of glycogen and lipids

The hepatic content of glycogen and lipids was determined in samples from groups G6. Glycogen was determined by the method detailed elsewhere [8]. The glucose in the supernatant before and after addition of amiloglucosidase was determined by enzymatic-colorimetric method (GoldAnalisa). Glycogen content was expressed as mmol glucose/g liver.

Liver lipids were extracted using chloroform and methanol [14]. Total lipids were measured by gravimetry and subsequently dissolved in chloroform and isopropanol (1:2). Triglycerides and cholesterol were determined with commercial kits (GoldAnalisa). Total lipid content was expressed as g lipid/g liver. Triglycerides and cholesterol were expressed as mg/g lipid.

### Western blot

Western blot analysis was performed as described before [15], with minor modifications. Samples from the liver of the G6 groups were homogenized in a lysis buffer and, after 40 min incubation, were centrifuged (12,000 g for 20 min at 4 °C) to obtain the protein extract. Total protein concentration was measured [16]. Protein samples of 30 μg were separated by 12% sodium dodecyl sulfate polyacrylamide gel electrophoresis. Proteins were then transferred onto nitrocellulose membranes, which were blocked, posteriorly, with Tris-buffered saline containing 5% BSA (wt/vol) for 1 h at room temperature. After blocking, membranes were incubated overnight at 4 °C with anti-phospho-FKHFser256 (dilution 1:500, sc-16,307, Santa Cruz Biotechnology, Dallas-TX, USA), anti-phospho-AMPKα (dilution 1:1000, #2535; Cell Signaling, Danvers-MA,USA), anti-PKA α/β/γ (dilution 1:500, sc-28,892, Santa Cruz Biotechnology), anti-PFK1 (dilution 1:500, sc-67,028, Santa Cruz Biotechnology) and anti-PEPCK (dilution 1:500, sc-32,879, Santa Cruz Biotechnology) primary antibodies. Protein bands were detected by chemiluminescence (SuperSignal™ West Fento; Pierce Biotechnology Inc., Rockford-IL, USA), after incubation with an appropriate horseradish peroxidase-conjugated secondary antibody and visualized using the C-DiGit® Blot Scanner (LI-COR Biosciences, Lincoln-NE, USA). Finally, band intensities were analyzed using ImageJ software (National Institutes of Health, Bethesda-MD, USA). The Ponceau S (P7179, Sigma Aldrich, St Louis-MO, USA) staining of the membrane was used as the loading control in these experiments [17].

### In situ liver perfusion

After overnight fasting, the rats were anesthetized with i.p. injection of thionembutal (40 mg/kg) after lidocaine (5 mg/kg) and subjected to cannulation of the portal vein and inferior cava vein. The liver was perfused in a non-recirculating system. The perfusion fluid (Krebs-Henseleit buffer, KH, pH 7.4) was pumped through an warmed (37 °C) membrane oxygenator saturated with O_2_/CO_2_ (95%/5%) before entering the liver through the portal vein at an estimated flux of 4 mL/min per g liver. Immediately after the beginning of the KH perfusion, the diaphragm was sectioned for euthanasia [5].

After 20 min of stabilization, samples of the effluent fluid were collected from the inferior cava vein every 5 min. During collection, the liver was sequentially perfused with KH buffer (basal perfusion, 10 min), KH buffer with glycogenolytic agent (adrenaline 1 μM, 30 min, ADR1 period), KH buffer with gluconeogenic substrate (lactate 2 mM, 30 min, LAC period) and again KH buffer with adrenaline 1 μM (30 min, ADR2 period). The concentrations of adrenaline and lactate were established in previous experiments.

From the samples of the effluent fluid it was determined the concentration of glucose (GoldAnalisa) and lactate [18]. The amount of each compound released by the liver (output) was expressed as μmol/min per g liver. The areas under the curve (AUCs) of the additional output of each compound were calculated taking the output at the end of the previous period as baseline. For basal perfusion, the AUCs of glucose were calculated as total output (baseline = 0). AUCs were expressed as μmol/g liver.

Body weight and naso-anal length of these animals were used to calculate the BMI (g/cm^2^). Inguinal, retroperitoneal, mesenteric and periepididymal fats were removed and weighed.

### Statistical analysis

Data were exhibited as mean ± SEM and were subjected to Shapiro-Wilk and Kolmogorov-Smirnov normality tests. Groups from the same litter size (G6 or G12) under the three feeding regimens (GL, GR and GRL) were compared through one-way ANOVA with Tukey’s post hoc test. Groups under the same feeding regimen (GL, GR or GRL) from the two litter sizes (G6 and G12) were compared in pairs through test t. The significance level adopted for all the comparisons was 5% (*p* < 0.05).

Statistical analysis and graphic construction were carried out in Prism® 5.0 (GraphPad, San Diego-CA, USA).

## Results

### Biometric records

At weaning, body weight of G12 was 30% lower than that of G6 (G6 = 56.49 ± 0.,82 g; G12 = 38.93 ± 0.94 g; *n* = 35–40/group, *p* < 0.05). The biometric data recorded at 90 d are shown in Table 1. It is evident the effect that the caloric restriction had on body weight, both in G6R and G12R, which were 45–49% less heavy than their corresponding GL (*p* < 0.05). Refeeding for 30 days partially reversed the effect of caloric restriction, which GRL weighing 60% more than their corresponding GR (*p* < 0.05), but still 10–20% less than the GL from the same litter size (*p* < 0.05).

**Table 1:** Biometric data of GL, GR and GRL rats

	G6L	G6R	G6RL	G12 L	G12R	G12RL
BW (g)	368.2 ± 7.18	193.9 ± 8.34^a^	335.6 ± 6.26^ab^	342.6 ± 9.72	182.6 ± 5.20^a^	293.1 ± 6.87^abc^
BMI (g/cm^2^)	0.58 ± 0.03	0.44 ± 0.01^a^	0.57 ± 0.01^b^	0.57 ± 0.01	0.44 ± 0.02^a^	0.55 ± 0.01^b^
RF (g%)	1.16 ± 0.14	0.23 ± 0.08^a^	0.90 ± 0.12^b^	0.86 ± 0.08	0.25 ± 0.05^a^	0.63 ± 0.05^ab^
MF (g%)	0.73 ± 0.05	0.31 ± 0.04^a^	0.50 ± 0.03^ab^	0.59 ± 0.06	0.29 ± 0.02^a^	0.45 ± 0.04^b^
PF (g%)	1.15 ± 0.03	0.79 ± 0.07^a^	1.03 ± 0.04^b^	0.92 ± 0.09	0.64 ± 0.07^a^	0.88 ± 0.05^bc^
VF (g%)	2.95 ± 0.11	1.33 ± 0.49^a^	2.30 ± 0.11^ab^	2.67 ± 0.14	1.19 ± 0.12^a^	1.96 ± 0.12^ab^
IF (g%)	0.90 ± 0.07	0.71 ± 0.07	0.87 ± 0.05	0.79 ± 0.04	0.54 ± 0.04^a^	0.87 ± 0.03^b^

Data shown as mean ± SEM, *n* = 6–8/group. ^a^ *p* < 0.05 vs GL of the same litter size; ^b^ *p* < 0.05 vs GR of the same litter size, *one-way* ANOVA/Tukey; ^c^ *p* < 0.05 vs G6 under the same feeding regimen, test t.

*BW* Body weight, *BMI* Body mass index, *RF* Retroperitoneal fat, *MF* Mesenteric fat, *PF* Periepididymal fat, *VF* Visceral fat, *IF* Inguinal fat

The BMI was also affected by caloric restriction, being approximately 25% lower in the GR from both litter sizes compared with their GL (*p* < 0.05). Refeeding completely restored BMI to GL values (*p* > 0.05).

As for differences related to litter size, only the body weight of G12RL was significantly lower than in G6RL (*p* < 0.05). The lower body weights in groups G12 L and G12R were not statistically different from G6L and G6R, respectively (*p* > 0.05).

Fat relative weights are also shown in Table 1. Retroperitoneal fat weight was 70–80% lower in the GR than in the GL (*p* < 0.05). Mesenteric fat was decreased by more than 50% because of caloric restriction in both litter sizes (G6R and G12R) compared with their corresponding GL (*p* < 0.05). The periepididymal fat weight had the same profile. The weight of these fats was greater after refeeding (GRL) than under caloric restriction (GR) (*p* < 0.05); fat weight recovery in comparison with GL was partial (*p* < 0.05) or complete (*p* > 0.05).

The summed weight of the visceral fats (retroperitoneal, mesenteric and periedididymal, Table 1) highlighted their significant decrease with caloric restriction and their partial recovery with refeeding.

The significantly less heavy peritoneal fat in the G12RL, compared with the G6RL (*p* < 0.05, Table 1), was the only difference due to litter size. The lower values of the other fats of the G12 did not differ statistically from the corresponding G6 (*p* > 0.05).

Inguinal fat weight was lower in the G12R than in the G12 L (*p* < 0.05), and once again refeeding was effective in restoring this value. The inguinal weight variation across the G6 was small and did not attain significance (*p* > 0.05).

### Biochemical determinations in blood and liver tissue

The values presented in Table 2 refer to fasting blood glucose and serum lipids. Blood glucose was higher in the GR than on the other groups (GL and GRL, *p* < 0.05), which were similar to each other. The comparison of litter size under the same feeding regimen did not show differences in fasting blood glucose (*p* > 0.05).

**Table 2:** Glucose (GLU), triglycerides (TG), total cholesterol (CHOL) and fractions of GL, GR and GRL rats

mg/dL	G6L	G6R	G6RL	G12 L	G12R	G12RL
GLU	80.0 ± 2.60	120.0 ± 4.78^a^	92.33 ± 3.46^b^	84.67 ± 2.86	107.5 ± 4.23^a^	94.5 ± 1.31^b^
TG	70.39 ± 1.71	68.29 ± 6.95	68.25 ± 3.71	66.58 ± 1.89	59.10 ± 2.40	60.00 ± 4.13
CHOL	79.99 ± 4.29	68.20 ± 2.77^a^	81.73 ± 2.54^b^	78.82 ± 2.29	70.10 ± 1.81^a^	75.56 ± 2.06
HDL	35.50 ± 2.74	43.40 ± 2.82	31.00 ± 4.25^b^	37.83 ± 2.44	46.90 ± 2.22^a^	30.42 ± 2.50^b^
VLDL	14.08 ± 0.34	14.63 ± 1.54	12.65 ± 0.74	13.32 ± 0.37	11.82 ± 0.48	12.00 ± 0.82
LDL	30.42 ± 2.49	11.12 ± 1.99^a^	37.08 ± 2.75^b^	27.67 ± 2.69	15.89 ± 1.45^a^	33.15 ± 1.53^b^

Data shown as mean ± SEM, *n* = 6/group. ^a^ *p* < 0.05 vs GL of the same litter size; ^b^ *p* < 0.05 vs GR of the same litter size, *one-way* ANOVA/Tukey

When comparing caloric-restricted groups (GR) with their respective freely-fed groups (GL), it was observed a significant decrease of total cholesterol (*p* < 0.05), which was matched by a decrease of the LDL fraction (*p* < 0.05). Refeeding increased LDL to values similar to (marginally higher, *p* > 0.05) the GL. The higher HDL values were significant when G12R was compared with G12L (*p* < 0.05). After refeeding (GRL), HDL was slightly lower (not significantly different, *p* > 0.05) than the corresponding GL. Triglycerides and VLDL did not differ across the groups (*p* > 0.05). Litter size did not influence the serum lipid profile (*p* > 0.05).

Liver glycogen and lipid content (Table 3) did not reveal statistically significant differences either due to feeding regimen or to litter size (*p* > 0.05 for all comparisons). Liver triglycerides and cholesterol were similar as well.

**Table 3:** Liver content of glycogen, total lipids, triglycerides and cholesterol of GR, GL and GRL rats

	G6L	G6R	G6RL
Glycogen (mmol/g liver)	0.48 ± 0.03	0.47 ± 0.04	0.51 ± 0.02
Total Lipids (g/100 g liver)	2.35 ± 0.50	2.39 ± 0.25	2.56 ± 0.05
Triglycerides (mg/g lipid)	72.32 ± 13.38	67.05 ± 8.43	57.23 ± 4.80
Cholesterol (mg/g lipid)	54.92 ± 15.17	43.90 ± 6.25	34.79 ± 3.37

Data shown as mean ± SEM, *n* = 4–6/group

### Liver output of glucose and lactate

**Fig. 2:**
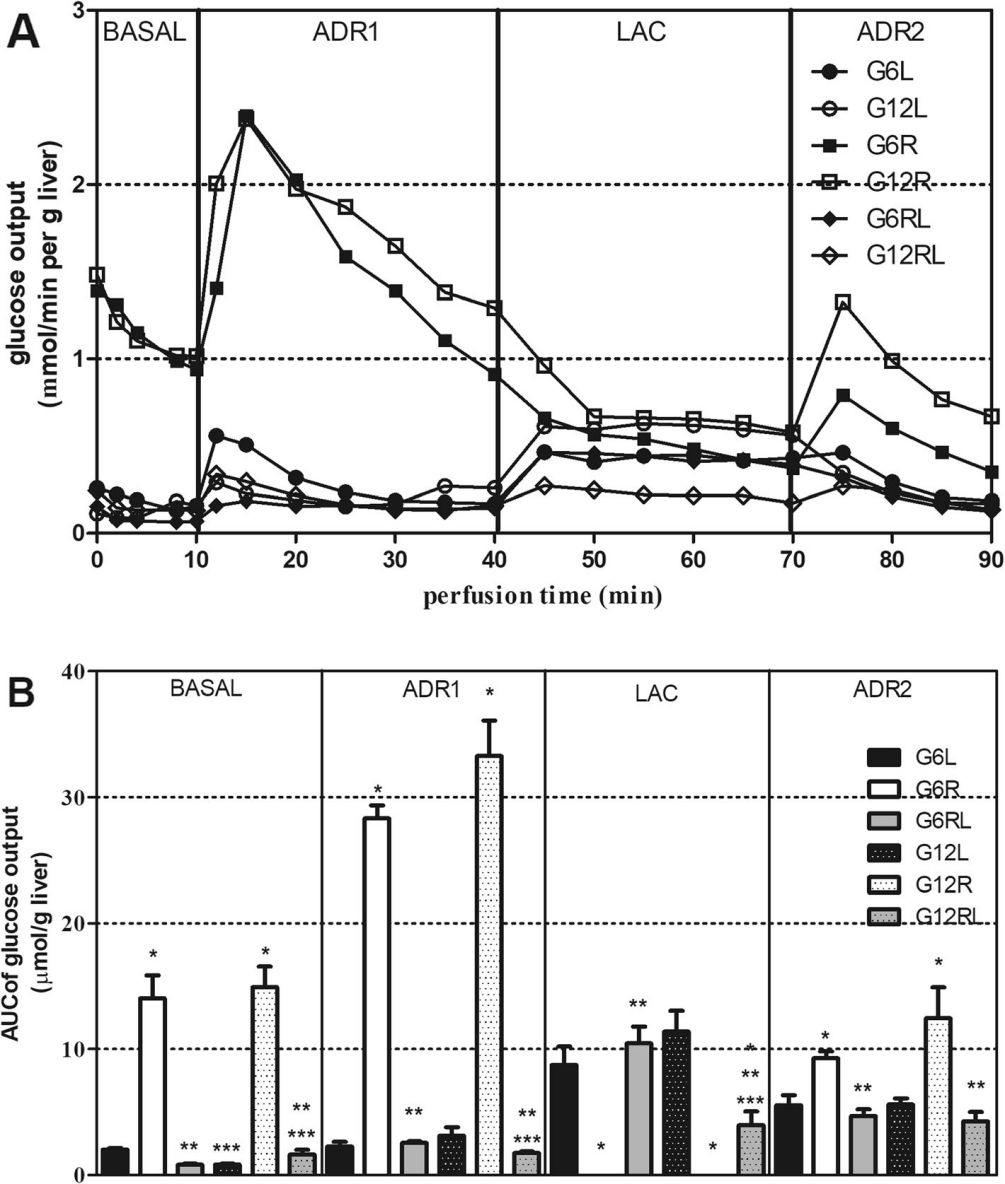
Glucose output during liver perfusion (**a**) and respective AUCs (**b**) of GL, GR and GRL rats. Data shown as mean (**a**) or mean ± SEM (**b**), *n* = 5–8/group. ^a^ *p* < 0.05 vs GL of the same litter size; ^b^ *p* < 0.05 vs GR of the same litter size, *one-way* ANOVA/Tukey; ^c^ *p* < 0.05 vs G6 under the same feeding regimen, test t

**Fig. 3:**
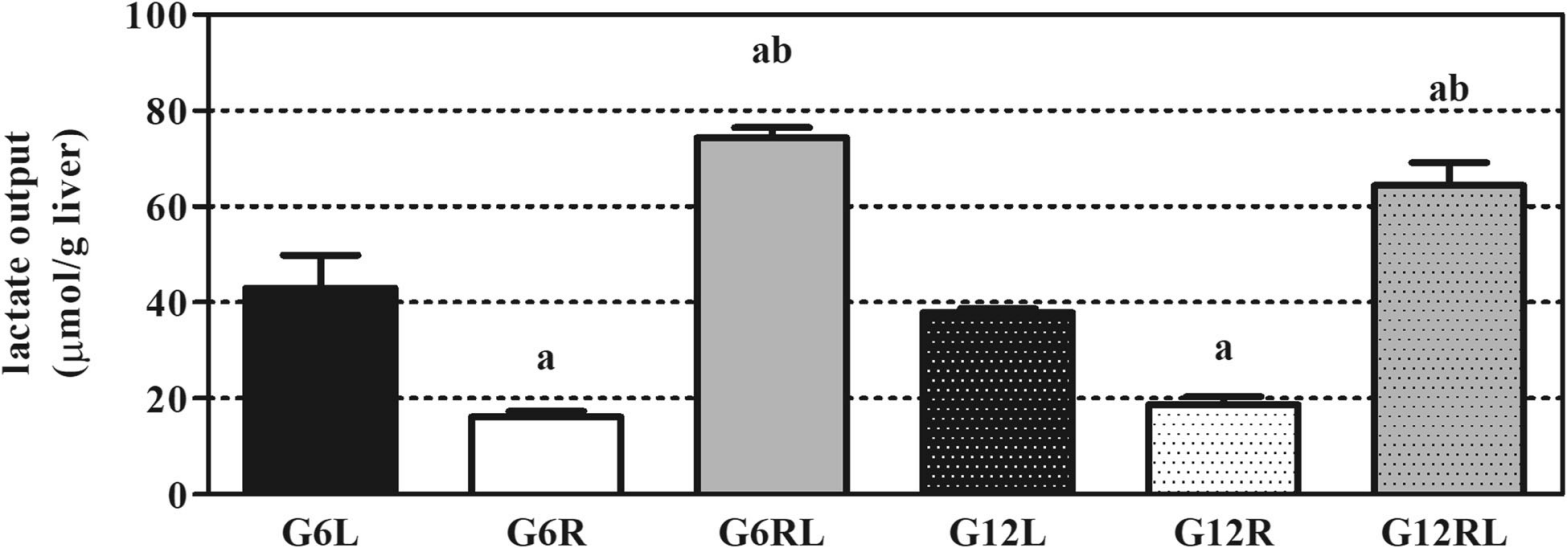
AUCs of lactate output during LAC period of liver perfusion of GL, GR and GRL rats. Data shown as mean ± SEM, *n* = 5–6/group. ^a^ *p* < 0.05 vs GL of the same litter size; ^b^ *p* < 0.05 vs GR of the same litter size, *one-way* ANOVA/Tukey

The effluent fluid collected during the 90-min perfusion allowed the analysis of glucose output in the absence or presence of compounds infused into the liver, dissolved in the KH buffer (Fig. 2a and b). Lactate output during the LAC period of perfusion was also determined (Fig. 3) to infer the liver uptake of this substrate.

Fig. 2a illustrates the mean glucose output of all the groups during the 90 min of in situ liver perfusion. The freely-fed groups, both G6L and G12L, and the refed groups (G6RL and G12RL) had a very low basal glucose output that was only slightly increased during adrenaline infusion (ADR1 and ADR2) but was elevated in the presence of lactate (LAC). This was in marked contrast with glucose output in the caloric-restricted groups, where total glucose output was high during the whole perfusion and adrenaline (ADR1 and ADR2) caused additional glucose output. On the other hand, lactate (LAC) did not enhance glucose output in these groups.

The AUCs in Fig. 2b represent the glucose outputs in each period of perfusion. In the basal period, the total glucose output was higher in GR (*p* < 0.05). The additional glucose output of these groups during ADR1 was more than 10 times higher when compared with the corresponding GL (*p* < 0.05). In the second adrenaline infusion (ADR2), glucose output in the caloric-restricted groups was lower than in the first (Fig. 2a), but once again was higher than in the other groups (*p* < 0.05). There were no differences between GL and GRL (*p* > 0.05), except between G12 L and G12RL during LAC (*p* < 0.05).

During lactate infusion (LAC), no additional glucose output was seen in the caloric-restricted groups (GR). On the other hand, the freely-fed (GL) and refed (GRL) groups increased glucose output in the presence of lactate.

A few statistical differences were recorded between litter sizes concerning the AUCs of glucose output during the periods of the liver perfusion; they are indicated in Fig. 2b. However, they were not consistent within a given feeding regimen and did not reveal a profile of glucose output (Fig. 2a) markedly different between litter sizes. The noticeable changes were between GR and their corresponding GL and GRL from the same litter size.

Fig. 3 has the AUCs of lactate output during LAC. Animals under caloric restriction (G6R and G12R) had lower output (*p* < 0.05), while the GRL had output values higher than the GL in both litter sizes (*p* < 0.05). Groups from the two litter sizes under the same feeding regimen had similar lactate output (*p* > 0.05).

### Expression of liver proteins

**Fig. 4:**
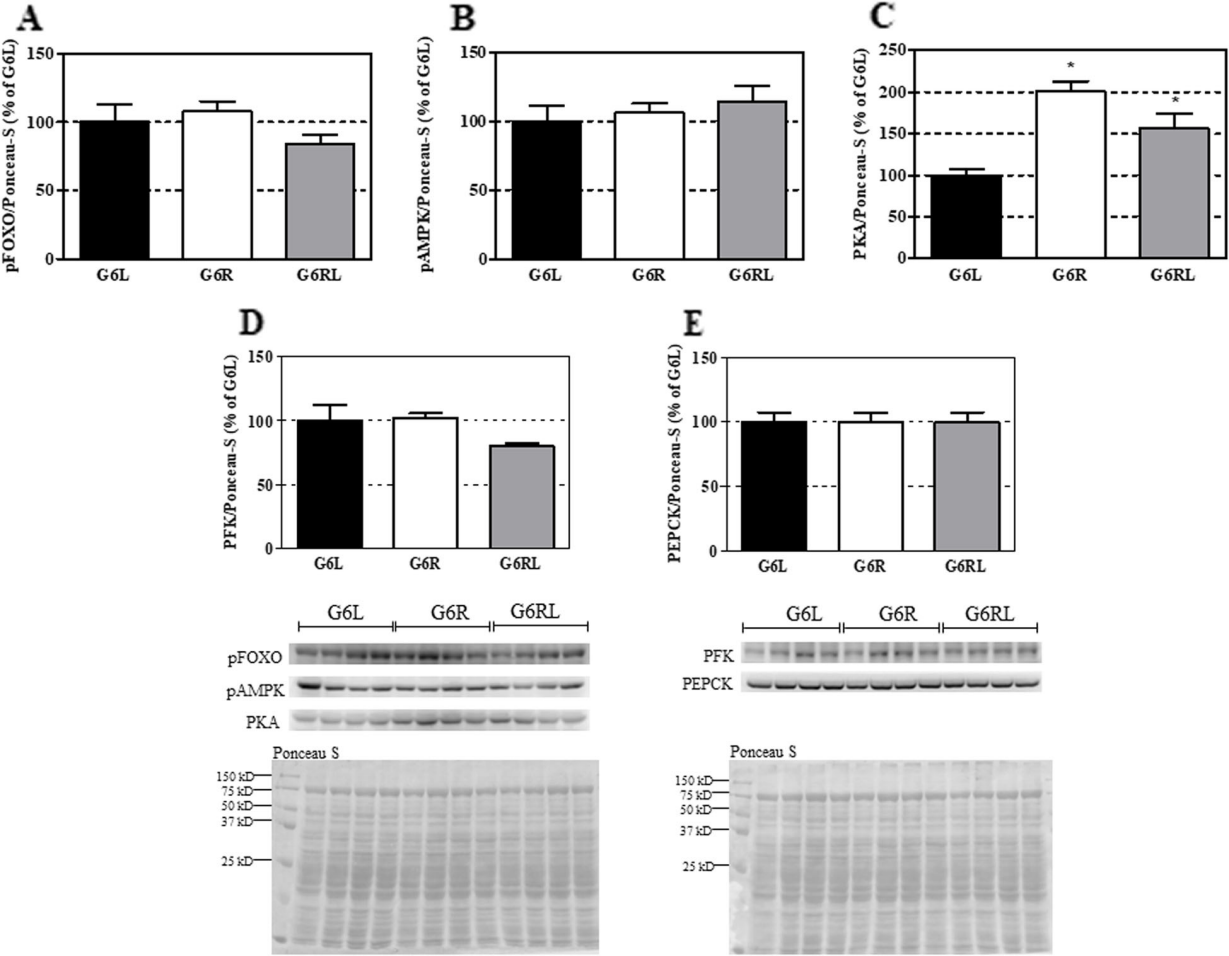
Expression of liver proteins of GL, GR and GRL rats. Data shown as mean ± SEM, *n* = 3–4/group. ^a^ *p* < 0.05 vs G6L, *one-way* ANOVA/Tukey

The expression of several G6 liver proteins related to energy metabolism – pAMPK, PFK, pFoxO and PEPCK – presented in Fig. 4, had insignificant changes across the groups. In contrast, a significant increase was recorded for PKA: 100% in G6R and 55% in G6RL in comparison with G6L (*p* < 0.05).

## Discussion

The changes across the groups of this study revealed a very clear pattern, primarily related to the feeding regimen when the assessments were made: caloric restriction caused biometric, biochemical and metabolic alterations that were totally or partially reversed upon refeeding.

Body weight and BMI of the caloric-restricted rats were quite consistent with previous observations by our research group, with decreased values because less calories were ingested since birth [7, 19]. The free supply of chow after the caloric restriction (GRL) promoted the recovery of these biometric values; the partial recovery of some of them was probably due to the short duration of refeeding (30 days).

The reduced visceral fat seems to have contributed importantly to lower body weight of the GR. In particular, the retroperitoneal and mesenteric fats decreased the most due to the caloric restriction but were restored upon refeeding from 60 d to 90 d, when positive energy balance was established. In contrast, the weight changes of the inguinal fat were small despite the feeding regimens. This contrast between visceral and subcutaneous (inguinal) fats can be linked to the higher visceral sensitivity to catecholamines, the circulating levels of which increase during fasting or stress [20], routine conditions for the caloric-restricted groups.

The feeding regimen also resulted in a consistent profile of blood values, with decreased total and LDL cholesterol and higher glucose with caloric restriction, and restoration to free-feeding values after refeeding. In other words, these parameters varied according to the nutritional condition, and the lipid profile benefited from caloric restriction, as reduced total and LDL cholesterol and higher HDL levels are correlated with decreased cardiovascular risk [21].

Glucose output during the 90 min of the in situ liver perfusion was much higher in the caloric-restricted groups than on those freely fed or refed. Fasting blood glucose of GR was higher as well. The possibility that the GR animals were in prandial or early post-prandial state can be discarded because all the animals were transferred to individual cages without chow or bedding material during the night before the experiments; in addition, the stomach and small intestine were empty and liver glycogen content was similar across de groups. The differences between GR and GL/GRL were, therefore, due to the chronic feeding regimen, and not to the fasting that preceded the experiment.

When lactate was given as gluconeogenic substrate, the additional glucose output was negligible in the GR. A similar observation was made with alanine [6, 8]. Curiously, lactate in the effluent fluid of the GR during this period (LAC) was the lowest, suggesting that lactate, which was given at equal rates to all the animals (about 10 μmol/min per g liver), was taken up more intensely by the liver of these animals. As this was not accompanied by an increased glucose output, the destination of the lactate in the GR hepatocytes cannot be unequivocally determined. Mitochondrial oxidation or gluconeogenesis followed by indirect glycogen synthesis [22] are two reasonable possibilities. On the other hand, the higher lactate output of GL and GRL accompanied by additional glucose output during LAC suggests that the substrate infusion was higher than the liver capacity of converting it to glucose for exportation. This reinforces the suggestion that, regardless of intracellular destination, lactate was more avidly taken up and used by the hepatocytes of the GR.

Another important point that was also observed in previous investigations of our group [5, 9] is that adrenaline, a glycogenolysis-stimulating hormone [23], promoted a large additional output of glucose in groups G6R and G12R. This output was more intense during the first infusion (ADR1), but also took place during ADR2. As caloric restriction kept these animals in post-prandial/fasting state during most of the time, facilitated liver glycogenolysis would be an important adaptative response for glucose homeostasis, and would also explain the higher fasting blood glucose of the GR.

This interpretation is consistent with the liver PKA expression, which had an increase of 100% in the G6R. PKA is in the center of the liver glycogenolytic action of two important counterregulatory hormones, glucagon and adrenaline [23, 24]. The PKA level in the G6R, twice as high as in G6L after overnight fasting, possibly indicates a higher liver sensitivity to these hormones, which would explain the high glucose output during the whole perfusion, the further increases in the presence of adrenaline (ADR1 and ADR2) and the higher fasting blood glucose of the GR, despite the equal glycogen content of the groups.

The alteration in the expression of PKA can be a measurable effect of the influence of caloric restriction on epigenetic mechanisms, such as DNA methylation, prevention of telomere shortening and chromatin protection. Such effects are often observed in research on this nutritional intervention, and it is believed that they are some of the mechanisms responsible for retardation of aging in animals subjected to caloric restriction [25, 26].

The increased expression of PKA in the refed group (G6RL, 55% compared with G6L) did not seem to be relevant to the point of promoting more glycogenolysis in the GRL than in the GL. Therefore, prolonged caloric restriction (GR) appears to modulate certain aspects of energy metabolism that refeeding (GRL) was capable of restoring completely after a relatively short period of 30 days (for instance, liver glucose output), while other variables may demand longer periods of refeeding after caloric restriction (for instance, body weight, visceral fat deposition and PKA expression) and hence were only partially recovered to GL levels after 30 days of refeeding.

The similar expression of pAMPK (a central element monitoring the cell energy status), PFK (a glycolytic enzyme), pFoxO (a transcription factor that activates gluconeogenic genes) and PEPCK (a key enzyme of gluconeogenesis) [23] across the groups suggests that these proteins were not responsive to the chronic feeding regimen. Rather, they were related to the immediate feeding state when the liver was collected – overnight fasting – and consequently were similar in all the groups. It can be speculated that, in a certain way, these GR animals adapted to the constant caloric restriction not only decreasing body growth and adiposity, but also regulating their liver energy metabolism at a new level.

It is improbable that the few differences between litter sizes have been caused, a priori, by the number of pups during lactation, or that these differences are biologically relevant within the context of this experimental model and the parameters (especially liver metabolism) that were investigated. Other factors, such as number of samples and inevitable oscillations on the conditions of maintenance, handling and experimentation of the animals, are more plausible explanations. Certainly, this reasoning does not apply to other morphological and functional variables in rodents from different litter sizes, as widely reported and discussed [10–12].

## Conclusions

From the standpoint of the hypothesis of this study, it was demonstrated that the liver changes due to caloric restriction and refeeding are not a residual effect of litter size and occur equally in rats from small (G6) and large (G12) litters. Therefore, the liver is responding to the current nutritional condition, and not to that from lactation. In addition, the increased expression of PKA points to a higher sensitivity of the animals under caloric restriction to glycogenolytic hormones, a relevant condition for glucose homeostasis during the prolonged fasting resulting from the limited amount of food.

## Data Availability

The datasets used and/or analyzed during the current study are available from the corresponding author on reasonable request.

## Abbreviations

CAPES: Coordination for the Improvement of Higher Education Personnel
CEUA: Ethics Commission on Animal Use
CONCEA: National Council of Control on Animal Experimentation
G12: Rats from 12-pups litters
G12 L and G6L: Rats from 12-pups and 6-pups litters fed freely from weaning until 90 days
G12R and G6R: Rats from 12-pups and 6-pups litters subjected to 50% caloric restriction from weaning until 90 days
G12RL and G6RL: Rats from 12-pups and 6-pups litters subjected to 50% caloric restriction from weaning to 60 days and fed freely until 90 days
GG: Glycogen
GLU: Glucose
KH: Krebs-Henseleit
pAMPK: phosphorylated AMP-dependent protein kinase
PEPCK: Phosphoenolpyruvate carboxy kinase
PFK: Phosphofructokinase
pFoxO: phosphorylated transcription factor forkhead box class O
PKA: cyclic AMP-dependent protein kinase
UEM: State University of Maringá
